# Racial and ethnic disparities in the association between financial hardship and self-reported weight change during the first year of the pandemic in the U.S.

**DOI:** 10.1186/s12939-023-02093-0

**Published:** 2024-01-22

**Authors:** Izabelle Mendez, Paula D Strassle, Erik J. Rodriquez, Stephanie Ponce, Randy Le, Alexis Green, Emma Martinez, Eliseo J Pérez-Stable, Anna M Nápoles

**Affiliations:** 1 Division of Intramural Research, National Institute on Minority Health and Health Disparities, National Institutes of Health, Bethesda, Maryland) USA; 2 Division of Intramural Research, National Heart, Lung and Blood Institute, National Institutes of Health, Bethesda, Maryland) USA; 3 Office of the Director, National Institute on Minority Health and Health Disparities, National Institutes of Health, Bethesda, Maryland) USA

**Keywords:** COVID-19, Weight change, Financial hardship, Food insecurity, Racial and ethnic minorities

## Abstract

Studies have shown that financial hardship can impact weight change; however, it is unclear what the economic impact of the COVID-19 pandemic has had on weight change in U.S. adults, or whether racial-ethnic groups were impacted differentially. We estimated the association between financial hardship and self-reported weight change using data from the cross-sectional COVID-19’s Unequal Racial Burden (CURB) survey, a nationally representative online survey of 5,500 American Indian/Alaska Native, Asian, Black/African American, Latino (English- and Spanish-speaking), Native Hawaiian/Pacific Islander, White, and multiracial adults conducted from 12/2020 to 2/2021. Financial hardship was measured over six domains (lost income, debt, unmet general expenses, unmet healthcare expenses, housing insecurity, and food insecurity). The association between each financial hardship domain and self-reported 3-level weight change variable were estimated using multinomial logistic regression, adjusting for sociodemographic and self-reported health. After adjustment, food insecurity was strongly associated with weight loss among American Indian/Alaska Native (aOR = 2.18, 95% CI = 1.05–4.77), Black/African American (aOR = 1.77, 95% CI = 1.02–3.11), and Spanish-speaking Latino adults (aOR = 2.32, 95% CI = 1.01–5.35). Unmet healthcare expenses were also strongly associated with weight loss among Black/African American, English-speaking Latino, Spanish-speaking Latino, and Native Hawaiian/Pacific Islander adults (aORs = 2.00-2.14). Other domains were associated with weight loss and/or weight gain, but associations were not as strong and less consistent across race-ethnicity. In conclusion, food insecurity and unmet healthcare expenses during the pandemic were strongly associated with weight loss among racial-ethnic minority groups. Using multi-dimensional measures of financial hardship provides a comprehensive assessment of the effects of specific financial hardship domains on weight change among diverse racial-ethnic groups

**Supplementary Information:**

The online version contains supplementary material available at 10.1186/s12939-023-02093-0.

## Introduction

Financial hardship disparities among racial and ethnic minority groups in the US have been exacerbated by the COVID-19 pandemic. For example, in June 2021, 23% of Asian, 38% of Latino, and 42% of Black adults were struggling to pay for household expenses compared to 21% of White adults [1]. Similarly, using data from our nationally representative survey of US adults, we found that American Indian/Alaska Native, Black/African American, English- and Spanish-speaking Latino, and Native Hawaiian/Pacific Islander adults were more likely to experience financial hardship during the first year of the pandemic, compared to White adults [2]. We also found that experiencing financial hardship across specific domains (e.g., food insecurity), was also more prevalent among racial and ethnic minorities [2]. 

Economic circumstances such as financial hardship may result in stress which is an important determinant for weight change [3, 4]. Experiencing stress can induce emotional eating, however, it can also suppress food intake, leading to variations on body weight changes such as weight gain or loss [5, 6]. Prior to the pandemic, having a lack of financial resources was associated with weight gain among British adults [7]. Furthermore, going hungry due to food insecurity was associated with weight loss among women in the US [8]. Weight gain (≥ 2.5-<10.0 kg) from early to middle adulthood may increase the risk of development of major chronic diseases (e.g., type 2 diabetes, cardiovascular disease, obesity-related cancers), and increased risk of morality among US adults compared with participants who maintained a stable weight [9]. Weight l loss of 5–10% of body weight in older adults was associated with an increase in all-cause mortality than those who had stable weight [10–12]. Additionally, significant weight gain or loss (> 10%) is associated with increased all-cause hospitalization compared to those with stable weight. Among racial and ethnic minorities, weight loss may be differentially associated with mortality. For instance, among older Japanese Americans and Latinos, weight loss had stronger associations with mortality than it did among Black/African American, Native Hawaiian, and White adults [13]. 

Maintaining stable weight at any level throughout adult life has been found to reduce mortality risk [14, 15]. Among individuals who are overweight or obese, weight stability was associated with improved subsequent clinical outcomes [16]. Weight loss in obese groups predicted a higher risk of all-cause mortality, which may be related to inappropriate dietary intake leading to a reduction in muscle mass rather than abdominal fat [17]. These studies indicates that weight stability and improving health habits, rather than weight loss or gain, may be a key health driver, especially for patients with obesity. Undesired weight change may also lead to repeated weight cycling (repeated loss and regain of weight) which is associated with increased risk for all-cause mortality regardless of BMI [18]. 

Despite the slow growing body of literature on the potential impact of financial hardship on weight change [7, 19], few studies have included racially and ethnically diverse populations or assessed whether the impact of financial hardship on weight change varied across racial-ethnic groups. Given racial and ethnic disparities in the burden of financial hardship in the US, the effects of financial hardship during a pandemic may vary across racial and ethnic groups. Furthermore, associations between various types of financial hardship and weight changes could differ by race and ethnicity. To prevent undesirable weight change in stressful times, it is necessary to investigate financial hardship and weight change. Thus, the purpose of our study was to (1) assess racial and ethnic differences in self-reported weight change during the COVID-19 pandemic and (2) estimate racial and ethnic specific associations between specific financial hardship domains and weight gain and weight loss during the first year of the pandemic among a nationally representative sample of Asian, Black/African American, Native Hawaiian/Pacific Islander, Latino (English- and Spanish-speaking), American Indian/Alaska Native, and White adults living in the US.

## Methods

### Study population and survey development

For this study, we used the cross-sectional COVID-19’s Unequal Racial Burden (CURB) online survey, a nationally representative survey of 1,000 Asian, 1,000 Black/African American, 1,000 Latino (500 Spanish-speaking), 1,000 White, 500 American Indian/Alaska Native, 500 Hawaiian/Pacific Islander, and 500 multiracial adults aged ≥ 18 years (*n* = 5,500 total) conducted between December 8, 2020, and February 17, 2021. The CURB survey was conducted in English and Spanish (Latino group only); details of the survey design and sampling strategy have been described elsewhere [20]. 

Briefly, the CURB survey was conducted by YouGov, which uses a proprietary opt-in panel (~ 1.8 million adults) to recruit survey participants. For this study, a target sample was generated using the 2018 American Community Survey 1-year sample data. Eligible participants from the panel were then proximity matched to the target sample on race and ethnicity, age, gender, education, and language preference (Latino sample only). Once recruitment quotas were met, sampling weights were then generated using multivariable logistic regression. Overall, this combination of matching and weighting allowed us to generate nationally representative samples within each racial and ethnic group (e.g., Asian participants were weighted to represent all Asian adults living in the United States). The National Institutes of Health Office of Institutional Review Board Operations determined that this study does not qualify as human subjects research because data used were de-identified (IRB# 000166).

### Self-reported weight change

All participants were asked, “How has your weight changed since the start of the pandemic?” with response options of lost a lot of weight, lost a little weight, weigh about the same, gained a little weight, or gained a lot of weight. These responses were then categorized into a 3-level weight change variable: (1) lost weight (little/a lot), (2) gained weight (little/a lot), and (3) no weight change [7, 21]. 

### Financial hardship during the COVID-19 pandemic

Financial hardship was assessed with twelve questions relating to economic and financial hardship during the pandemic, the majority of which were adapted from the All of Us COVID-19 Participant Experience (COPE) survey [2]. Using these 12 questions, we identified six different domains of financial hardship which capture the loss of employment and the potential cascade of effects due to lost wages: (1) lost income, (2) debt, (3) unmet expenses (general needs), (4) unmet healthcare expenses, (5) housing insecurity, and (6) food insecurity. If a participant responded ‘yes’ to any of the domain-specific questions, they were flagged as having experienced that financial hardship (e.g., lost income). Lost income domain was measured using two questions; “Since the start of the pandemic, have you lost your job or business?” and “Since the start of the pandemic, have you lost any work-related income?”. The debt domain was measured using the following 2 questions “Since the start of the pandemic, have you had to use up all or most of your savings?” and “Since the start of the pandemic, have you gone into debt or has your debt increased?”. Unmet expenses (general needs) domain were measured using 2 questions; “Since the start of the pandemic, was there ever a time when you did not have enough money to meet your daily needs?” and “Since the start of the pandemic, was there ever a time when you did not have enough money to pay your monthly bills?”. Unmet healthcare expenses domain were measured using 3 questions; “Since the start of the pandemic, was there ever a time when you lost your health insurance?”, “Since the start of the pandemic, was there ever a time when you did not have enough money to pay for the health care that you needed?” and “Since the start of the pandemic, was there ever a time when you did not have enough money to pay for your medications?”. The housing insecurity domain was measured using 2 questions “Since the start of the pandemic, was there ever a time when you did not have enough money to pay your rent, mortgage, or other housing costs?” and “Since the start of the pandemic, was there ever a time when you did not have a regular place to live?”. Food insecurity was measured using 1 question, “Since the start of the pandemic, was there ever a time when you were hungry but didn’t eat because there wasn’t enough money for food?”. A more detailed description of the financial hardship questions and creation of the financial hardship domains and index have been published elsewhere [2]. 

### Race and ethnicity and other participant characteristics

All participants were asked “Which one of the following would you say best represents your race and ethnicity?” Response options were Latino/a/x or Hispanic, American Indian or Alaska Native, Asian, Black or African American, Pacific Islander, White, and multiracial. Latino participants were further stratified into English- and Spanish-speaking based on their survey language preference; 87.6% of Latino participants who took the survey in Spanish also self-reported poor English-speaking skills (compared to only 12.4% among those who took the survey in English).

Other covariates of interest were selected based on prior literature examining financial hardship, which included gender (male, female, and transgender/non-binary), age (18–39, 40–59, and 60 + years), marital status (married, unmarried), income (<$20,000, $20,000-$59,999, $60,000-$99,999, ≥$100,000) highest education level (less than high school graduate, high school graduate, some college/vocational school, and college graduate or more), self-reported physical health (poor/fair versus good/very good/excellent), and chronic conditions [7]. Participants were flagged as having a chronic condition if they reported having ever been diagnosed with chronic obstructive pulmonary disease (COPD), heart conditions (such as heart failure, coronary artery disease, or cardiomyopathies), type 2 diabetes, chronic kidney disease or on dialysis, sickle cell disease, cancer in the past year, or being immunocompromised due to a transplant. Participants were also asked if a medical doctor had ever told them they were obese. All participant characteristics were self-reported.

### Statistical analyses

Chi-square tests were used to compare the prevalence of weight loss and weight gain among those who experienced and did not experience each financial hardship domain, overall and stratified by race and ethnicity. Multinomial logistic regression, adjusting for age, gender, marital status, income, highest education level, self-reported physical health, and chronic conditions (yes/no), to estimate associations between financial hardship and weight change (weight gain and weight loss vs. no change [reference group]). Separate models were run within each racial and ethnic group to estimate race and ethnicity-specific associations. Multiracial group were not included in the analysis due to the limited interpretability of those included in this category. A single model with race-ethnicity and an interaction term between race and ethnicity and financial hardship was also used to assess whether the effects financial hardship on weight changes varied across race and ethnicity prior to stratification. Because results were consistent across approaches, only the findings from the stratified models are presented. All six financial domains were included simultaneously in the models to estimate the independent association between each domain and weight change. Due to small cell sizes, transgender and non-binary adults were excluded from modeling.

All analyses were performed in SAS version 9.4 (SAS Inc., Cary, NC) and were weighted to produce nationally representative estimates within each racial-ethnic group. Counts were rounded for interpretation.

## Results

### Participant characteristics

Sociodemographics, overall and among those who experienced any financial hardship, are reported in Tables 1 and 2. Among participants who experienced “any” financial hardship during the first year of the pandemic, Spanish-speaking Latino participants were more likely than other groups to report being uninsured (54.3% vs. 18.1–26.4%) and having less than a high school education (30.6% vs. 4.7–13.7%), Table 2. Black/African American and Spanish-speaking Latino participants experiencing financial hardship were more likely than other groups to report having a family income <$20,000 (39.4% and 38.0% vs. 17.9–33.2%). Overall, the most common financial hardship domains were debt (57.6%), lost income (44.5%), and unmet expenses (33.7%), Supplemental Table 1. Data on racial and ethnic disparities in the prevalence of financial hardship domains has been published elsewhere [2]. 

**Table 1:** Participant characteristics, stratified by race-ethnicity, CURB survey, *N* = 5,500

		American Indian/ Alaska Native	Asian	Black/African American	English- Speaking Latino	Spanish-Speaking Latino	Native Hawaiian/ Pacific Islander	White	Multiracial
**Total, N**		500	1000	1000	500	500	500	1000	500
**Age, median (IQR)**		43 (27.0)	38 (20.0)	41 (27.0)	38 (27.0)	37 (17.0)	37 (21.0)	51 (27.0)	36 (24.0)
**Gender, n (%)**									
Male		231 (46.2)	456 (45.9)	472 (47.2)	217 (43.7)	273 (54.3)	236 (47.2)	475 (47.6)	228 (45.6)
Female		245 (49.1)	525 (52.8)	513 (51.3)	268 (54.1)	224 (44.4)	250 (50.0)	509 (50.9)	238 (47.5)
Transgender or non-binary^a^		24 (4.8)	13 (1.3)	15 (1.5)	11 (2.3)	7 (1.3)	14 (2.8)	15 (1.5)	35 (6.9)
**Fair/Poor Physical health, n (%)**		181 (36.1)	256 (25.6)	256 (25.6)	137 (27.7)	105 (20.9)	143 (28.7)	243 (24.3)	163 (32.6)
**Chronic condition(s)**^**b**^, **n (%)**		160 (32.0)	181 (18.1)	223 (22.3)	102 (20.5)	77 (15.2)	117 (23.5)	214 (21.4)	78 (15.7)
**Obesity, n (%)**		102 (20.3)	114 (11.4)	141 (14.1)	99 (20.0)	53 (10.6)	77 (15.5)	194 (19.4)	80 (16.1)
**Health insurance, n (%)**									
Any private insurance		157 (31.6)	589 (59.0)	342 (34.4)	196 (39.5)	110 (22.4)	198 (39.8)	540 (54.0)	252 (50.5)
Public insurance only		238 (47.7)	260 (26.0)	431 (43.3)	181 (36.6)	122 (24.9)	212 (42.6)	337 (33.7)	172 (34.6)
Uninsured		103 (20.8)	149 (15.0)	222 (22.3)	118 (23.9)	259 (52.7)	88 (17.6)	122 (12.2)	74 (14.9)
**Immigration status, n (%)**									
US-born citizen		485 (97.2)	515 (51.5)	907 (90.7)	408 (82.3)	86 (17.1)	443 (88.7)	977 (97.8)	456 (91.1)
Foreign-born citizen/legal resident		14 (2.8)	433 (43.3)	83 (8.3)	77 (15.6)	224 (44.5)	50 (10.1)	21 (2.1)	43 (8.6)
Undocumented		0(0.0)	52 (5.2)	10 (1.0)	11 (2.2)	194 (38.4)	6 (1.2)	1 (0.1)	1 (0.3)
**Education, n (%)**									
Less than high school graduate		57 (11.4)	40 (4.0)	68 (6.8)	41 (8.3)	146 (29.1)	41 (8.3)	55 (5.5)	49 (9.8)
High school/GED		192 (38.5)	229 (22.9)	375 (37.5)	175 (35.3)	212 (42.1)	196 (39.2)	291 (29.1)	120 (24.0)
Some college/vocational school		181 (36.2)	215 (21.5)	351 (35.1)	174 (35.2)	99 (19.7)	178 (35.7)	312 (31.2)	180 (36.0)
College graduate or more		70 (13.9)	516 (51.6)	207 (20.7)	105 (21.3)	46 (9.1)	84 (16.8)	342 (34.2)	151 (30.1)
**Family annual income**^**c**^, **n (%)**									
<$20,000		123 (26.7)	106 (12.4)	292 (33.3)	93 (21.0)	154 (35.3)	110 (24.8)	130 (15.2)	86 (19.8)
$20,000-$59,000		193 (41.9)	281 (32.8)	346 (39.4)	199 (45.1)	224 (51.3)	165 (37.1)	336 (39.2)	177 (41.0)
$60,000-$99,000		81 (17.5)	229 (26.7)	137 (15.6)	96 (21.8)	43 (9.8)	108 (24.3)	181 (21.1)	100 (23.1)
≥$100,000		64 (13.9)	241 (28.1)	103 (11.8)	53 (12.1)	16 (3.6)	61 (13.7)	211 (24.6)	69 (16.0)
**Married/ partnership, n(%)**		238 (47.6)	507 (50.7)	318 (31.8)	220 (44.4)	313 (62.1)	248 (49.6)	522 (52.2)	186 (37.2)
**Household Configuration, n (%)**									
Lives alone		78 (15.7)	173 (17.3)	240 (24.0)	72 (14.4)	46 (9.1)	81 (16.1)	198 (19.8)	90 (18.1)
Adults only		252 (50.4)	516 (51.6)	389 (38.9)	253 (51.0)	142 (28.2)	209 (41.9)	580 (58.0)	253 (50.5)
Single parent/children only		22 (4.3)	30 (3.0)	86 (8.6)	19 (3.8)	25 (4.9)	15 (3.0)	19 (1.9)	13 (2.7)
Adults and children		148 (29.6)	280 (28.0)	285 (28.5)	153 (30.8)	291 (57.8)	195 (39.0)	204 (20.4)	144 (28.7)
**Residence urbanicity, n (%)**									
Big city		99 (19.9)	265 (26.5)	344 (34.9)	152 (31.0)	223 (45.0)	98 (19.5)	130 (15.1)	122 (24.3)
Smaller city		92 (18.4)	161 (16.1)	193 (19.6)	108 (21.9)	159 (31.6)	89 (17.7)	134 (15.5)	87 (17.4)
Suburban area		101 (20.2)	415 (41.6)	300 (30.4)	155 (31.4)	74 (14.7)	153 (30.7)	285 (32.9)	182 (36.3)
Small town		94 (18.8)	106 (10.6)	83 (8.4)	38 (7.7)	29 (5.7)	98 (19.6)	150 (17.4)	62 (12.3)
Rural		114 (22.8)	53 (5.3)	67 (6.8)	39 (8.0)	17 (3.4)	62 (12.5)	166 (19.2)	48 (9.5)

^a^ Includes individuals who identified as non-binary, gender queer, gender fluid, other, and none

^b^ Chronic conditions included: cancer, chronic obstructive pulmonary disease (COPD), chronic kidney disease, diabetes, heart conditions, immunocompromised from transplant, and sickle cell disease

^c^ Collected by YouGov at enrollment into panel

**Table 2:** Participant characteristics, among those who reported experiencing any financial hardship^a^ stratified by race-ethnicity

	American Indian/ Alaska Native	Asian	Black/African American	English-Speaking Latino	Spanish-Speaking Latino	Native Hawaiian/ Pacific Islander	White	Multiracial
**Total, N**	384	586	765	364	440	393	580	350
**Age, median (IQR)**	42 (27.0)	38 (19.0)	40 (26.0)	37 (25.0)	36 (16.0)	36 (19.0)	47 (26.0)	36 (23.0)
**Gender, n (%)**								
Male	172 (44.7)	257 (43.9)	356 (46.6)	153 (41.9)	246 (56.0)	179 (45.5)	254 (43.9)	159 (45.3)
Female	194 (50.6)	319 (54.4)	398 (52.1)	202 (55.4)	187 (42.5)	203 (51.7)	314 (54.2)	165 (47.0)
Transgender or non-binary^a^	18 (4.7)	10 (1.7)	10 (1.4)	10 (2.7)	7 (1.5)	11 (2.7)	11 (1.9)	27 (7.7)
**Fair/poor physical health, n (%)**	150 (39.0)	179 (30.5)	224 (29.3)	121 (33.2)	94 (21.5)	124 (31.6)	167 (28.9)	132 (37.8)
**Chronic condition(s)**^**b**^, **n (%)**	123 (32.0)	116 (19.8)	168 (22.0)	82 (22.5)	68 (15.4)	96 (24.3)	124 (21.4)	59 (16.7)
**Obesity, n (%)**	89 (23.2)	84 (14.4)	110 (14.3)	82 (22.6)	43 (9.7)	60 (15.3)	128 (22.0)	63 (18.0)
**Health insurance, n (%)**								
Any private insurance	98 (25.7)	294 (50.3)	224 (29.3)	124 (34.3)	88 (20.5)	136 (34.7)	256 (44.2)	139 (39.9)
Public insurance only	193 (50.2)	174 (29.8)	349 (45.7)	142 (39.2)	108 (25.2)	174 (44.5)	219 (37.8)	146 (41.7)
Uninsured	93 (24.1)	117 (20.0)	191 (25.0)	96 (26.4)	234 (54.3)	81 (20.8)	105 (18.1)	64 (18.4)
**Immigration status, n (%)**								
US-born citizen	371 (96.9)	294 (50.2)	697 (91.2)	302 (83.1)	73 (16.5)	346 (88.3)	565 (97.6)	327 (93.3)
Foreign-born citizen/legal resident	12 (3.1)	265 (45.3)	61 (8.0)	53 (14.5)	196 (44.7)	41 (10.5)	13 (2.2)	22 (6.3)
Undocumented	0(0.0)	26 (4.5)	7 (0.9)	9 (2.4)	170 (38.8)	5 (1.2)	1 (0.1)	1 (0.4)
**Education, n (%)**								
Less than high school graduate	52 (13.7)	27 (4.7)	60 (7.8)	33 (9.1)	134 (30.6)	36 (9.1)	43 (7.3)	35 (9.9)
High school/GED	154 (40.2)	160 (27.3)	290 (37.9)	129 (35.4)	185 (42.0)	158 (40.3)	176 (30.4)	88 (25.2)
Some college/vocational school	136 (35.5)	141 (24.1)	283 (37.0)	135 (37.0)	84 (19.1)	140 (35.5)	187 (32.2)	128 (36.5)
College graduate or more	41 (10.6)	258 (43.9)	132 (17.3)	67 (18.5)	36 (8.3)	59 (15.1)	175 (30.1)	100 (28.4)
**Family annual income**^**c**^,**n (%)**								
<$20,000	119 (33.2)	89 (17.9)	270 (39.4)	77 (23.7)	145 (38.0)	103 (29.4)	110 (21.6)	78 (25.3)
$20,000-$59,999	153 (42.9)	187 (37.5)	268 (39.0)	162 (50.0)	196 (51.4)	139 (39.4)	217 (42.5)	126 (41.0)
$60,000-$99,999	59 (16.6)	126 (25.3)	102 (14.9)	60 (18.5)	30 (8.0)	73 (20.7)	98 (19.2)	71 (23.0)
≥$100,000	26 (7.2)	96 (19.3)	46 (6.6)	25 (7.8)	10 (2.6)	37 (10.5)	86 (16.8)	33 (10.7)
**Married/ partnership, n (%)**	171 (44.6)	271 (46.3)	226 (29.5)	154 (42.2)	266 (60.5)	186 (47.4)	266 (45.8)	129 (36.9)
**Household configuration, n (%)**								
Lives alone	64 (16.6)	123 (21.0)	179 (23.4)	48 (13.1)	39 (9.0)	58 (14.7)	118 (20.4)	63 (18.1)
Adults only	184 (48.1)	271 (46.3)	269 (35.1)	173 (47.6)	120 (27.3)	153 (38.9)	300 (51.7)	171 (48.8)
Single parent/child(ren) only	17 (4.5)	23 (3.9)	77 (10.1)	15 (4.3)	23 (5.1)	14 (3.5)	15 (2.5)	10 (2.8)
Adults and children	118 (30.8)	169 (28.9)	240 (31.3)	128 (35.0)	258 (58.6)	169 (42.9)	147 (25.4)	106 (30.2)
**Residence urbanicity, n (%)**								
Big city	86 (22.5)	153 (26.2)	254 (33.7)	114 (31.8)	195 (44.5)	73 (18.6)	93 (17.7)	86 (24.6)
Smaller city	75 (19.5)	94 (16.0)	167 (22.2)	88 (24.6)	136 (31.1)	75 (19.2)	85 (16.2)	68 (19.3)
Suburban area	66 (17.3)	222 (38.0)	206 (27.4)	101 (28.2)	63 (14.5)	116 (30.0)	151 (28.9)	118 (33.7)
Small town	73 (18.9)	72 (12.2)	70 (9.3)	31 (8.7)	26 (6.0)	77 (20.0)	97 (18.7)	41 (11.6)
Rural	83 (21.7)	45 (7.6)	56 (7.4)	24 (6.7)	17 (3.9)	51 (12.9)	97 (18.5)	38 (11.0)

Any financial hardship status during the first year of the pandemic was categorized as dichotomous (≥ 1 [any] vs. no hardships)

^a^ Includes individuals who identified as non-binary, gender queer, gender fluid, other, and none

^b^ Chronic conditions included: cancer, chronic obstructive pulmonary disease (COPD), chronic kidney disease, diabetes, heart conditions, immunocompromised from transplant, and sickle cell disease

^c^Collected by YouGov at enrollment into panel

### Prevalence of self-reported weight

**Fig. 1:**
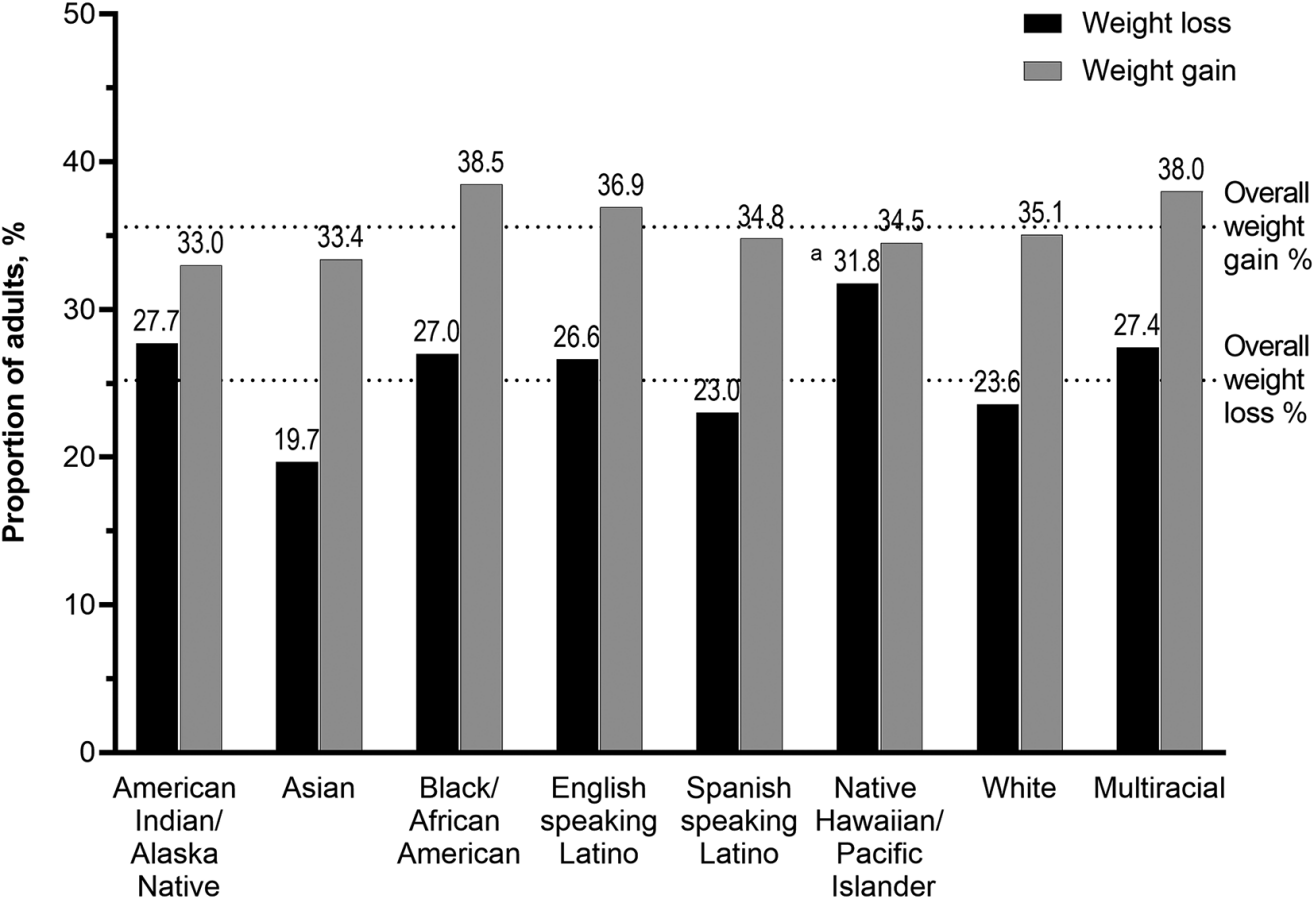
Prevalence of self-reported weight loss and weight gain during the first year of the pandemic, stratified by race-ethnicity, December 2020-Februrary 2021.^a^ Significance level of p = < 0.0001 for Chi squared test

Overall, 25.2% of participants reported losing weight (lost a lot: 7.2%; lost a little: 18.0%) and 35.6% reported gaining weight (gained a lot: 9.2%; gained a little: 26.4%) during the pandemic, Fig. 1 and Supplemental Table 2. Native Hawaiian/Pacific Islander adults (31.8% *p* = 0.0004) were most likely to report weight loss, followed by American Indian/Alaska Native (27.7%), multiracial (27.4%), and Black/African American (27.0%) adults. At least one-third of participants in each racial and ethnic group reported weight gain during the first year of the pandemic (33.0-38.5%).

### Prevalence of financial hardship and weight change among racial and ethnic groups

**Fig. 2:**
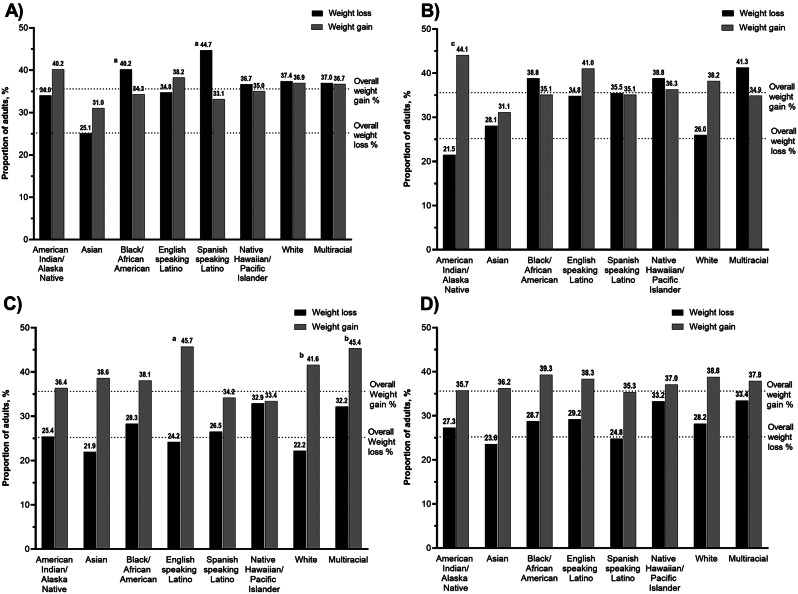
Prevalence of weight gain and weight loss among those who experienced (**A**) Food insecurity, (**B**) unmet healthcare expenses, (**C**)lost income, or (**D**) debt, during the first year of the pandemic, stratified by race-ethnicity, December 2020-Februrary 2021. Significance level of ^a^*p* < 0.0001, ^b^*p* < 0.01, ^c^*p* < 0.05 for Chi squared test

When examining the prevalence of weight change among those who did experience specific financial hardship domains, racial and ethnic differences were seen. For example, among Spanish-speaking Latino (44.7% p = < 0.0001) and Black/African American (40.2%, p = < 0.0001) adults who experienced food insecurity, they were more likely to report weight loss compared to other racial and ethnic groups (vs. 25.1-37.4%), Fig. 2A. Among American Indian/Alaska Native (44.1%, *p* = 0.02) and English-speaking Latino adults (41.0%) who experienced unmet healthcare expenses were more likely to report weight gain compared to other racial and ethnic groups (vs. 31.1%-38.2), Fig. 2B. Furthermore, among White (41.6%, *p* = 0.002), multiracial (45.4%, *p* = 0.003), and English-speaking Latino (45.7%, *p* = 0.0005) adults who experienced lost income were more likely to report weight gain compared to other racial-ethnic groups (vs. 33.4-38.6%), Fig. 2C. Among those who experienced debt, the prevalence in weight gain were similar across racial and ethnic groups (35.7-39.3%), Fig. 2D.

### Associations between Financial hardship and weight loss

After adjusting for sociodemographics and self-reported health, overall, the financial hardship domains: unmet healthcare expenses (aOR = 1.36, 95% CI = 1.12–1.67), debt (aOR = 1.25, 95% CI = 1.03–1.51), and food insecurity (aOR = 1.37, 95% CI = 1.06–1.77) were significantly associated with weight loss. When stratifying among racial and ethnic groups, differences in the associations between financial hardship and weight loss also persisted. For example, food insecurity was associated with weight loss among American Indian/Alaska Native (aOR = 2.18, 95% CI = 1.05–4.77), Black/African American (aOR = 1.77, 95% CI = 1.02, 3.11), and Spanish-speaking Latino adults (aOR = 2.32, 95% CI = 1.01, 5.35), Table 3. Experiencing unmet healthcare expenses was associated with weight loss among Black/African American (aOR = 2.10, 95% CI = 1.29–3.19), English-speaking Latino (aOR = 2.14, 95% CI = 1.06, 4.72), and Native Hawaiian/Pacific Islander adults (aOR = 2.00, 95% CI = 1.03, 3.82). Experiencing unmet expenses (general) was associated with weight loss among Asian adults (aOR = 1.82, 95% CI = 1.02, 3.36) adults.

**Table 3:** Adjusted race-ethnicity specific associations between experiencing financial hardship domains and weight loss

	Lost Income^b^	Unmet expenses^c^	Debt^d^	Unmet healthcare expenses^e^	Housing insecurity^f^	Food insecurity^g^
	OR (95% CI)^a^	OR (95% CI)^a^	OR (95% CI)^a^	OR(95% CI)^a^	OR (95% CI)^a^	OR (95% CI)^a^
Overall	1.07(0.90, 1.27)	1.07(0.87, 1.34)	**1.25 (1.03, 1.51)**	**1.36 (1.12, 1.67)**	1.17 (0.93, 1.48)	**1.37 (1.06, 1.77)**
American Indian/Alaska Native	1.16 (0.65, 2.07)	0.50 (0.24, 1.00)	1.32 (0.69, 2.55)	0.61 (0.31, 1.19)	1.57 (0.71, 3.47)	**2.18 (1.05, 4.77)**
Asian	1.07 (0.71, 1.63)	**1.82 (1.02, 3.63)**	1.03 (0.63, 1.69)	1.26 (0.73, 2.19)	0.70 (0.35, 1.41)	0.88 (0.38, 2.00)
Black/ African American	0.85 (0.56, 1.29)	1.47 (0.91, 2.37)	0.92 (0.58, 1.46)	**2.10 (1.29, 3.19)**	1.31 (0.78, 2.18)	**1.77 (1.02, 3.11)**
English-speaking Latino	1.14 (0.61, 2.13)	**0.26 (0.12, 0.60)**	1.61 (0.84, 3.07)	**2.14 (1.06, 4.72)**	1.59 (0.67, 3.75)	1.47 (0.58, 3.68)
Spanish-speaking Latino	1.92 (0.86, 4.27)	1.78 (0.88, 3.60**)**	0.92 (0.37, 2.25)	1.57 (0.85, 2.90)	0.85 (0.44, 1.62)	**2.32 (1.01, 5.35**)
Native Hawaiian/Pacific Islander	1.00 (0.57, 1.76)	1.07 (0.51, 2.26)	1.81 (0.96, 3.41)	**2.00 (1.03, 3.82)**	0.63 (0.29, 1.37)	1.20 (0.58, 2.49)
White	0.95 (0.60, 1.50)	1.34 (0.69, 2.49)	1.60 (0.99, 2.61)	0.82 (0.44, 1.51)	1.77 (0.87, 3.63)	1.37 (0.72, 3.20)

Abbreviations: aOR, adjusted odds ratio; CI, confidence interval

^a^ Adjusted for age, gender (male, female), highest education level, income, marital status, chronic conditions (yes/no) and self-reported physical health; ORs were estimated using multinomial logistic regression where outcome was specified as weight loss, weight gain, or no change [reference group]; weight gain results are presented in Table 4. Separate models were run for each racial-ethnic group

*Note:* Bold font denote statistical significance p < 0.05

^b^ Lost income included loss of job or reduced hours, or loss of work-related income

^c^ Unmet expenses included not having enough money to meet daily needs or not enough money to pay monthly bills

^d^ Debt included using up all/most of savings, having no savings before the pandemic, or having gone into debt or increased debt during the pandemic

^e^ Unmet healthcare expenses included loss of health insurance, not having enough money to pay for healthcare, and not having enough money to pay for medications

^f^ Housing insecurity included not having a regular place to live and not having enough money to pay rent, mortgage, or housing costs

^g^ Food insecurity included being hungry but didn’t eat because not enough money for food

Associations between Financial hardship and weight gain:

After adjusting for sociodemographics and self-reported health, overall, the financial hardship domain: lost income (aOR = 1.32, 95% CI = 1.10–1.50), was significantly associated with weight gain Table 4. Among the racial and ethnic groups, lost income was associated with weight gain among Asian adults (aOR = 1.63, 95% CI = 1.12, 2.29). Unmet expenses were also associated with weight gain among Spanish-speaking Latino (aOR = 2.29, 95% CI = 1.25, 4.23) and Native Hawaiian/Pacific Islander adults (aOR = 3.09, 95% CI = 1.49, 6.63).

**Table 4:** Adjusted race-ethnicity specific associations between experiencing financial hardship domains and weight gain

	Lost Income^b^	Unmet expenses^c^	Debt^d^	Unmet healthcare expenses^e^	Housing insecurity^f^	Food insecurity^g^
	OR (95% CI)^a^	OR (95% CI)^a^	OR (95% CI)^a^	OR(95% CI)^a^	OR (95% CI)^a^	OR (95% CI)^a^
Overall	**1.32 (1.10, 1.50)**	1.15 (0.94, 1.41)	1.19 (1.00, 1.46)	1.13 (0.94, 1.37)	0.95 (0.76, 1.18)	1.02 (0.79, 1.31)
American Indian/Alaska Native	1.13 (0.65, 1.96)	0.64 (0.33, 1.24)	0.99 (0.53, 1.87)	1.42 (0.77, 2.61)	1.36 (0.77, 2.61)	1.67 (0.81, 3.45)
Asian	**1.62 (1.12, 2.29)**	0.73 (0.42, 1.28)	1.42 (0.93, 2.16)	0.95 (0.57, 1.58)	1.64 (0.89, 3.03)	0.72 (0.33, 1.59)
Black/ African American	1.23 (0.84,1.77)	0.94 (0.61, 1.45)	1.25 (0.83, 1.89)	1.20 (0.77, 1.86)	1.08 (0.67, 1.74)	1.17 (0.68, 2.02)
English-speaking Latino	1.72 (0.98, 3.02)	0.64 (0.32, 1.29)	1.29 (0.70, 2.37)	1.89 (0.96, 3.73)	0.98 (0.46, 2.11)	0.91 (0.40, 2.11)
Spanish-speaking Latino	1.08 (0.58, 2.01)	**2.29 (1.25, 4.23)**	1.20 (0.59, 2.46)	1.25 (0.71, 2.20)	**0.43 (0.24, 0.76)**	2.05 (0.90, 4.67)
Native Hawaiian/Pacific Islander	0.73 (0.42, 1.28)	**3.09 (1.49, 6.63)**	1.10 (0.57, 2.13)	1.63 (0.83, 3.19)	0.46 (0.21, 1.00)	0.63 (0.30, 1.33)
White	1.43 (0.90, 1.82)	1.49 (0.87, 2.41)	1.47 (0.92, 2.18)	0.75 (0.46, 1.21)	1.38 (0.64, 2.22)	0.80 (0.48, 1.93)

Abbreviations: aOR, adjusted odds ratio; CI, confidence interval

^a^ Adjusted for age, gender (male, female), highest education level, income, marital status, chronic conditions (yes/no) and self-reported physical health; ORs were estimated using multinomial logistic regression where outcome was specified as weight loss, weight gain, or no change [reference group]; weight loss results are presented in Table 3 Separate models were run for each racial-ethnic group

**Note:** Bold font denote statistical significance p < 0.05

^b^ Lost income included loss of job or reduced hours, or loss of work-related income

^c^ Unmet expenses included not having enough money to meet daily needs or not enough money to pay monthly bills

^d^ Debt included using up all/most of savings, having no savings before the pandemic, or having gone into debt or increased debt during the pandemic

^e^ Unmet healthcare expenses included loss of health insurance, not having enough money to pay for healthcare, and not having enough money to pay for medications

^f^ Housing insecurity included not having a regular place to live and not having enough money to pay rent, mortgage, or housing costs

^g^ Food insecurity included being hungry but didn’t eat because not enough money for food

## Discussion

In a diverse nationally representative sample of U.S. adults, we found that among all participants, 25.2% reported experiencing weight loss and 35.6% reported weight gain during the first year of the pandemic. The prevalence of weight loss varied across racial and ethnic groups, with Native Hawaiian/Pacific Islander adults being the most likely racial and ethnic group to report weight loss, followed closely by Black/African American, and English-speaking Latino adults. Regarding weight loss, minoritized racial and ethnic groups (American Indian/Alaska Native, Black/African American, English-and Spanish-speaking Latinos and Native Hawaiian/Pacific Islander) were more vulnerable from exposure to the Financial hardship domains: food insecurity and unmet healthcare expenses. Among those reporting weight gain, minoritized racial and ethnic groups (Asian, English-speaking Latino and Spanish-speaking Latino) were more vulnerable from exposure to the Financial hardship domains; lost income and unmet expenses (general). Overall, minoritized racial and ethnic groups were more vulnerable to financial hardship burdens and weight change during the first year of the pandemic.

Although there are few studies that examine the relationship among financial hardship and weight change in the US, our findings are relatively consistent with international studies which found that weight change may depend on the type of hardship experienced [22]. For instance, one study found that financial hardship (enough money for needs, frequency of not enough money, and difficulty paying bills) had a differential impact on weight change among older British men and women, while another study found that persistent insufficient money for food/clothing was associated with weight gain only in British women [7, 22]. And while studies of race and ethnicity specific associations between financial hardship domains and weight change are limited, in a previous analyses, when examining age associations we found that the effects of financial hardship during the COVID-19 pandemic may impact weight change differentially across age, specifically weight loss [23]. 

In our study, we found that food insecurity was strongly associated with weight loss among American Indian/Alaska Native, Black/African American, and Spanish-speaking Latino adults. Prior to the pandemic, American Indian/Alaska Native communities have been found to experience widespread food insecurity [24]. Furthermore, an analysis examining trends in food insecurity from 2001 to 2016 found that rates for both Black/African American and Latino households were at least twice that of white households [25]. During the COVID-19 pandemic, a study found that Black, Latinos, and other racial minority adults were more likely to experience food insecurity compared to White and Asian adults in the United States [26]. In our study, severe food security was measured, and at severe levels of food insecurity, many adults who report being hungry because there was not enough money, experience reduce food intake and often report not eating for an entire day [27]. Vulnerable minority racial-ethnic populations who are at higher risk of food insecurity may experience periods of deprivation and/or underconsumption of food which may lead to weight loss [28, 29]. 

We also found that experiencing unmet healthcare expenses was associated with weight loss among Black/African American, English-speaking Latino, and Native Hawaiian/Pacific Islander adults. Native Hawaiian/Pacific Islander and Latino adults are less likely to have health insurance coverage than White adults [30, 31]. Similar to Latino adults, Native Hawaiian/Pacific Islander adults may have limited English proficiency which could restrict access to health insurance [31, 32]. Latino and Black/African American adults are more often employed in low-wage jobs that do not provide adequate health insurance, leaving them with higher out-of-pocket expenses [33]. Consistent with our results, we found that among 33.3% Black/African American reported earning less than 20,000$ and 50% of English-speaking Latino adults reported earning between $20,000-$59,999. Although there is a lack of literature that examines the relationship between weight loss and unmet healthcare expenses, lack of health insurance often results in poorer health outcomes, such as reduced quality of life, premature death, and poor diet quality [34]. Additionally, lack of health care coverage is a surrogate marker for undiagnosed comorbidities [35]. Undiagnosed diseases may lead to unintentional weight loss; for example, poor glycemic control in undiagnosed diabetes may lead to involuntary weight loss [36, 37]. 

Among Asian, adults, lost income was associated increased odds of weight gain. During the pandemic, racial and ethnic minority groups, particularly Asian adults, experienced negative impacts on employment compared to White adults [38]. An increase in anti-Asian sentiments in the United States also led to increased labor market discrimination against Asian Americans [38]. Prior to the pandemic, a British study found that job loss and lost income were associated with increased likelihood of weight gain due to changes in diet, sleep, physical activity, and smoking [39]. 

In summary, we found that specific types of financial hardship (food insecurity, unmet healthcare expenses, lost income) were associated with weight change among marginalized racial and ethnic communities. These findings may be indicative of the long-term effects of structural racism and discrimination on the ability of minority households to access resources and handle economic shocks. For example, Black/African American adults with the same income and education as White adults continue to have less overall wealth (e.g., equity on a home, savings) in the US [40]. For Spanish-speaking Latino populations in the United States, many are foreign-born and are more likely to report financial hardships due to fewer resources, such as lower median incomes and higher levels of poverty, than native-born households [41, 42]. For American Indian/Alaska Native communities, historically tribes were forcibly removed and relocated from their ancestral lands and placed on reservations and allotments with limited access to resources and infrastructure (e.g., roads, electricity, water, broadband) [24]. This concentration of social and economic disadvantage in marginalized groups leads to disparities in food insecurity, healthcare coverage, and healthcare access among these communities, and was further exacerbated during the COVID-19 pandemic [2, 33, 43]. 

This study has a few limitations. First, the YouGov survey was a cross-sectional survey which cannot be used for causal associations and to analyze behavior over a period to time. It was also administered online, and adults with limited internet access or familiarity with technology are less likely to be represented. Furthermore, our survey was only administered in English and Spanish for Latino participants only, potentially excluding non-Spanish speaking individuals with limited English proficiency. Furthermore, we acknowledge the monolithic classification of Asians, which may continue the perpetuation of the model minority myth that all Asians are well-educated and affluent, inappropriately masks the enormous health disparities within diverse Asian groups. Weight change was made based upon self-report rather than physical exam measure; however, at least one study has found that using weight change introduced minimal measurement error and found that there was little difference between self-report and measured weight change [44–46]. Furthermore majority of our sample reported a little weight loss or weight gain, and were unable to quantify weight change to determine its clinical significance. We are also unable to determine whether the weight loss or weight gain was unintentional or due to purposeful changes in diet and exercise. Unintentional weight loss (involuntary decline in total body weight over time) may reflect disease severity (e.g., in patients with advanced heart disease, lung disease or malignant disease) or undiagnosed illness [47], both of which may have worsened with mitigation measures implemented in response to the COVID-19 pandemic. There we also no measure to determine starting weight, diet, diet quality, food deserts, and resource utilization such as SNAP to adjust for in our models. We acknowledge that someone who has low food security may not have any weight change because the limited food that they can access is high in sugar and/or calories but low in nutritional value (i.e., inexpensive junk food, fast food, sodas, etc.). Additionally, the measures of financial hardship are not comprehensive. For example, we asked if individuals had ‘lost [their] job or business’ but did not ask about other household or family members, which could also cause hardship. We are also unable to determine if financial hardship was experienced prior to the COVID-19 pandemic or caused by the pandemic.

In conclusion, our findings suggest that financial hardship during the pandemic was associated with weight change, among minoritized racial and ethnic minority groups. Given the association between (unintentional) weight loss, morbidity, and mortality, it is critical to continue to assess the potential long-term consequences of the COVID-19 pandemic, weight change and financial hardship among these communities. While financial hardship was prevalent among minoritized racial and ethnic groups in our study, at this timepoint the US government provided economic relief in response to the pandemic. There were historic expansions for financial aid (e.g., SNAP benefits, unemployment benefits, the pandemic electronic benefit transfer (P-EBT) program, etc.) [48]. Given the expansion for these financial programs, data would need to be collected on financial hardship in the post-pandemic era. These programs may have played a significant role in reducing food insecurity pre-pandemic, however, it is possible that it was not nearly sufficient to ameliorate the economic impacts exacerbated due to the pandemic among minority communities. Our results highlight the type of financial hardship domains that interventions or programs can target among minoritized communities such as unmet healthcare expenses and food insecurity. Furthermore, interventions, public health practitioners, and healthcare professionals should promote weight stability and healthy coping mechanisms to avoid weight change during stressful times. Lastly, using multi-dimensional measures of financial hardship provides a comprehensive assessment of the effects of specific financial hardship domains on weight change among diverse racial and ethnic groups.

### Electronic supplementary material

Below is the link to the electronic supplementary material.

Supplementary Material 1: Supplementary figures and tables

## Data Availability

Available upon request.

## Code Availability

Available upon request.
