# Supplementary material for: Racial and ethnic disparities in the association between financial hardship and self-reported weight change during the first year of the pandemic in the U.S
Source: Int J Equity Health. 2024 Jan 22;23:12. doi: 10.1186/s12939-023-02093-0 (PMC10804602; doi:10.1186/s12939-023-02093-0)
Supplement: Supplementary file 1 — Supplementary Material 1: Supplementary figures and tables [file 12939_2023_2093_MOESM1_ESM.docx]

**Supplemental Figure 1**. : Prevalence of weight gain and weight loss among those who experienced financial hardship domains, during the first year of the pandemic, December 2020-Februrary 2021.


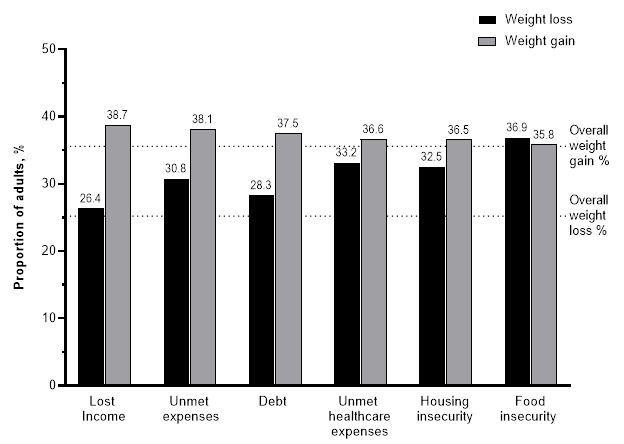


**Supplemental Table 1**. Prevalence of financial hardship by domain during the first year of the pandemic, stratified by race-ethnicity and overall, December 2020-Februrary 2021.

|  | **Lost Income^a^** | **Unmet expenses^b^** | **Debt^c^** | **Unmet Healthcare expenses^d^** | **Housing insecurity^e^** | **Food insecurity^f^** |
| --- | --- | --- | --- | --- | --- | --- |
|  | **N (%)** | **N (%)** | **N (%)** | **N (%)** | **N (%)** | **N (%)** |
| Overall | 2,448 (44.5) | 1,854 (33.7) | 3,165 (57.6) | 1,250 (22.7) | 1,008 (18.3) | 707 (12.9) |
| American Indian/Alaska Native | 223 (44.6) | 189 (37.8) | 328 (65.5) | 143 (28.6) | 110 (22.0) | 102 (20.4) |
| Asian | 393 (39.3) | 194 (19.4) | 385 (38.5) | 176 (17.6) | 103 (10.3) | 56 (5.6) |
| Black/African American | 428 (42.9) | 419 (41.9) | 643 (64.3) | 247 (24.7) | 215 (21.5) | 157 (15.7) |
| English-speaking Latino | 210 (42.4) | 180 (36.3) | 322 (64.9) | 115 (23.1) | 92 (18.5) | 62 (12.5) |
| Spanish-speaking Latino | 368 (73.1) | 269 (53.3) | 406 (80.7) | 144 (28.6) | 155 (30.8) | 66 (13.1) |
| Native Hawaiian/Pacific Islander | 259 (51.9) | 221 (44.7) | 332 (66.4) | 137 (27.6) | 136 (27.4) | 102 (20.5) |
| White | 349 (34.9) | 226 (22.6) | 464 (46.4) | 162 (16.2) | 109 (10.9) | 90 (9.0) |
| Multiracial | 217 (43.4) | 156 (31.2) | 285 (57.1) | 126 (25.3) | 89 (17.8) | 72 (14.5) |

^a^ Lost income included loss of job or reduced hours, or loss of work-related income

^b^ Unmet expenses included not having enough money to meet daily needs or not enough money to pay monthly bills

^c^ Debt included using up all/most of savings, having no savings before the pandemic, or having gone into debt or increased debt during the pandemic

^d^ Unmet healthcare expenses included loss of health insurance, not having enough money to pay for healthcare, and not having enough money to pay for medications

^e^ Housing insecurity included not having a regular place to live and not having enough money to pay rent, mortgage, or housing costs

^f^ Food insecurity included being hungry but didn’t eat because not enough money for food

**Supplemental Table 2**. Prevalence of self-reported weight change during the first year of the pandemic, overall and stratified by race-ethnicity, December 2020-Februrary 2021.

|  | **Lost a lot** | **Lost a little** | **No change** | **Gained a little** | **Gained a lot** |
| --- | --- | --- | --- | --- | --- |
|  | **N (%)** | **N (%)** | **N (%)** | **N (%)** | **N (%)** |
| Overall | 393 (7.2) | 993 (18.0) | 2,158 (39.2) | 1,450 (26.4) | 506 (9.2) |
| American Indian/Alaska Native | 32 (6.4) | 107 (21.3) | 196 (39.3) | 118 (23.6) | 47 (9.4) |
| Asian | 47 (4.7) | 150 (15.0) | 469 (46.9) | 269 (26.9) | 65 (6.5) |
| Black/ African American | 100 (10.0) | 170 (17.0) | 345 (34.5) | 264 (26.4) | 121 (12.1) |
| English-speaking Latino | 37 (7.4) | 91 (18.3) | 181 (36.5) | 123 (24.7) | 60 (12.2) |
| Spanish-speaking Latino | 23 (4.5) | 93 (18.5) | 212 (42.2) | 142 (28.1) | 34 (6.7) |
| Native Hawaiian/Pacific Islander | 47 (9.5) | 112 (22.3) | 168 (33.7) | 118 (23.5) | 55 (11.0) |
| White | 70 (7.0) | 166 (16.6) | 413 (41.3) | 281 (28.1) | 70 (7.0) |
| Multiracial | 33 (6.6) | 104 (20.9) | 173 (34.6) | 136 (27.1) | 54 (10.9) |
